# Structural analysis of traditional Chinese complex puzzle locks

**DOI:** 10.1038/s41598-022-15326-z

**Published:** 2022-07-04

**Authors:** Kan Shi, Ming Jie Wang, Yang Zhang, Kuo Hung Hsiao, Yan An Yao

**Affiliations:** 1 College of Mechanical and Electronic Engineering, Shandong University of Science and Technology, Qingdao, 266590 China; 2 College of Mechanical and Electrical Engineering, Beijing University of Chemical Technology, Beijing, 100029 China; 3 Collections and Research Division, National Science and Technology Museum, Kaohsiung, 807044 Taiwan; 4 School of Mechanical, Electronic and Control Engineering, Beijing Jiaotong University, Beijing, 100044 China

**Keywords:** Engineering, Mechanical engineering

## Abstract

The development of barbed-spring locks in ancient China has a history of more than 2000 years. With the development of the design and manufacturing techniques in ancient China, the safety of locks has gotten better and better. Since the seventeenth century, the puzzle lock, with a complicated structure and a high difficulty in opening, was gradually developed and used. The puzzle lock needs specific steps to be opened. Based on the difficulty of the opening process, traditional Chinese puzzle locks can be partition into two sorts, namely, general puzzle locks and complex puzzle locks. As the structure of the puzzle lock will change during the opening process, the puzzle lock belongs to the reconfigurable mechanism. In this paper, a method of topology matrix is provided to analyze the structure of the complex puzzle lock during systematical operation. Firstly, the characteristics and types of general puzzle locks are explained, and then the topology matrix representation is introduced. Finally, four complex puzzle locks are taken as examples, to illustrate the opening process. There are various types of complex puzzle locks, and the mechanism designs are quite ingenious and interesting, which shows the extraordinary technique and ingenuity of the ancient craftsmen.

## Introduction

Locks have been used in ancient China for more than two thousands of years and are skillful mechanical devices. However, they were made by locksmiths of a humble status, who were almost unknown. Thus, the records on ancient locks in literature are lacking. Since ancient times, locksmiths have used different materials, such as wood, bronze, brass and iron, to make locks. Therefore, studying the historical development and design of locks can reflect the development of contemporary technology, in order to gain a better understanding of the social, cultural and economic development at that time^1^. Furthermore, locks have special designs, use different ideas that strengthen the internal structure and appearance of the design, and complex puzzle locks are developed with an extremely high opening degree of difficulty, and their safety is better than that of ordinary locks. These complex puzzle locks were even educational toys for ancient literati, scholars, officials, and aristocrats to play with and to show off. The mechanism of a puzzle lock displays the mechanical design and manufacturing technique, and it has a high preservation and research value. However, due to negligence, the number of existing puzzle locks in ancient China is gradually decreasing, and the speed of damage and loss is accelerating day by day.

In modern times, many experts, scholars and research units have devoted themselves to researching ancient locks. Needham concisely presented the development of locks in China and the West, discussed their mutual influence, described the inner structure of some representative locks, sighed for the shortage of literature on the evolution of locks in China, which needed more attention and research, and mentioned that it would be unforgivable to ignore the deeds of locksmiths^2^. Since 1990, Yan has collected and studied ancient Chinese locks and systematically introduced their development^3,4^. In addition, Yan et al. provided a design methodology of keys, keyholes, bolts, and spring configurations of ancient Chinese padlocks^5–7^. Yates combed archaeological documents and discovered that the earliest complete barbed-spring lock came from the site of the Qin Emperor Mausoleum, which is more than 2200 years old^8^. Zhou et al. took ancient locks as examples to introduce the historical evolution and characteristics of barbed-spring locks^9^. Liger analyzed many ancient lock relics and various forms of European barbed-spring locks that were unearthed^10^. Pitt-Rivers collected a large number of ancient locks, and analyzed and discussed the form, communication and evolution of locks in the world^11^. Hommel introduced familiar wooden locks in China and compared them with those used in the countrified regions of France, Germany and Italy^12^. Tanavoli presented the history and the types of ancient Iranian locks, which is an important document for studying ancient Iranian locks^13^. Rasmussen et al. conducted a field investigation and oral interviews with old locksmiths to discuss the historical development and types of traditional Chinese padlocks, and the rich content provided important reference data for studying traditional Chinese locks^14^. Shi et al. discussed the structure of existing Chinese labyrinth locks and provided that the procedure of inserting a key head into the keyhole can be considered as the corresponding relationship between two members and a pair^15^. Hsiao collected and discussed the types of locks with open and hidden keyholes in ancient China, and systematically analyzed their mechanical structure^16,17^. Shi et al. collected and studied Chinese wooden locks, discussed the features and structure of wooden locks, and put forward the analysis results of ten types of ancient wooden locks^18^. Millington introduced the early combination locks around the world, explained the design implication of the combination lock, and pinpointed the opening mode through the internal structure^19^. Zhang et al. discussed the structure and characteristics of barbed-spring locks and put forward the manufacturing mode of traditional Chinese locks^20^. Ceccarelli provided a way of innovative design for the study of ancient mechanical devices^21^. Ye carefully combed the Chinese lock culture and collected hundreds of Chinese lock pictures, which provided valuable materials for collectors^22^.

**Figure 1:**
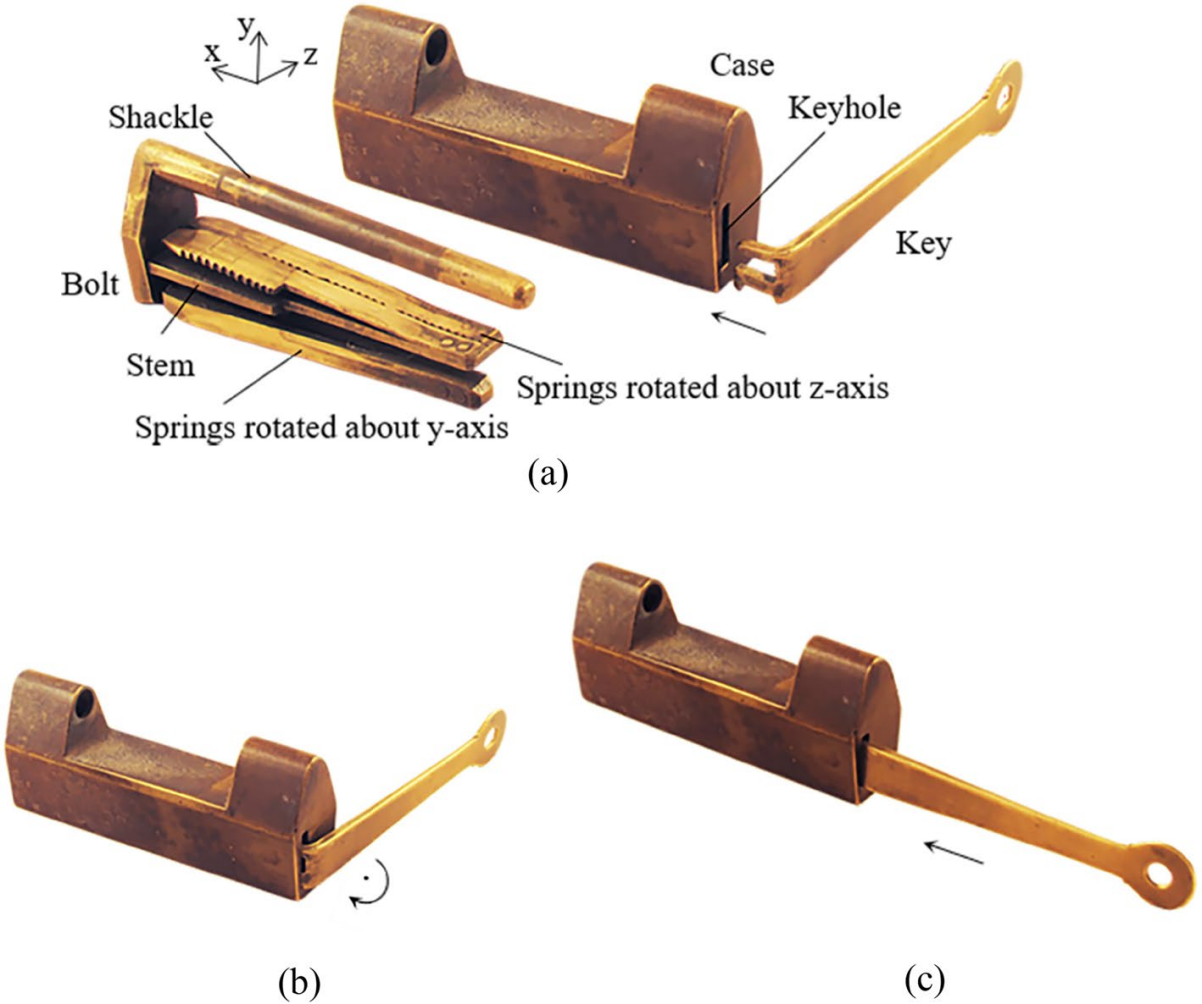
Barbed-spring lock.

Figure 1a shows a simple barbed-spring lock. As the keyhole can be seen directly from the case, it can also be called an open-keyhole lock, which was a common type of lock in ancient China. It consists of the case, the bolt with the shackle and the stem, the barbed-spring (spring for short), and the key. The case has a keyhole for inserting the key and guiding the bolt to move. The bolt has a shackle for suspending the lock and a stem for riveting the springs^23^. The key is designed according to the position and shape of the keyhole and the configuration of the spring. When locking, due to the action of an elastic force, and the springs are stuck in the inner wall of the case. When unlocking, the key head squeezes the springs to move the springs out of the inner wall, and then moves the bolt by the key, to separate the bolt from the case, to complete the unlocking.

Chinese puzzle locks are endowed with cultural meaning and technique, showing the creativity and ingenuity of ancient craftsmen with regard to their mechanical design. The purpose of this work is to analyze the structure of traditional Chinese complex puzzle locks, to explain briefly the types and characteristics of puzzle locks, and to provide the topology matrix representation of locks. Finally, four complex puzzle locks are taken as examples, to illustrate their proposed representation.

## Types and characteristics of general puzzle locks

Most of the ancient locks in other parts of the world only need to insert the key into the keyhole and unlock it by rotating or sliding the key. However, for traditional Chinese puzzle locks, people often hold the correct key in their hands, but need to use their minds or even rack their brains on how to insert the key into the keyhole. Some puzzle locks need to use different keys or go through several specific steps to complete the unlocking procedure, which is quite distinctive for different lock types. According to different design methods and opening steps, general puzzle locks can be sorted into four types: additional obstacle locks, maze locks, two-section locks, and hidden-keyhole locks, which are introduced below.

**Figure 2:**
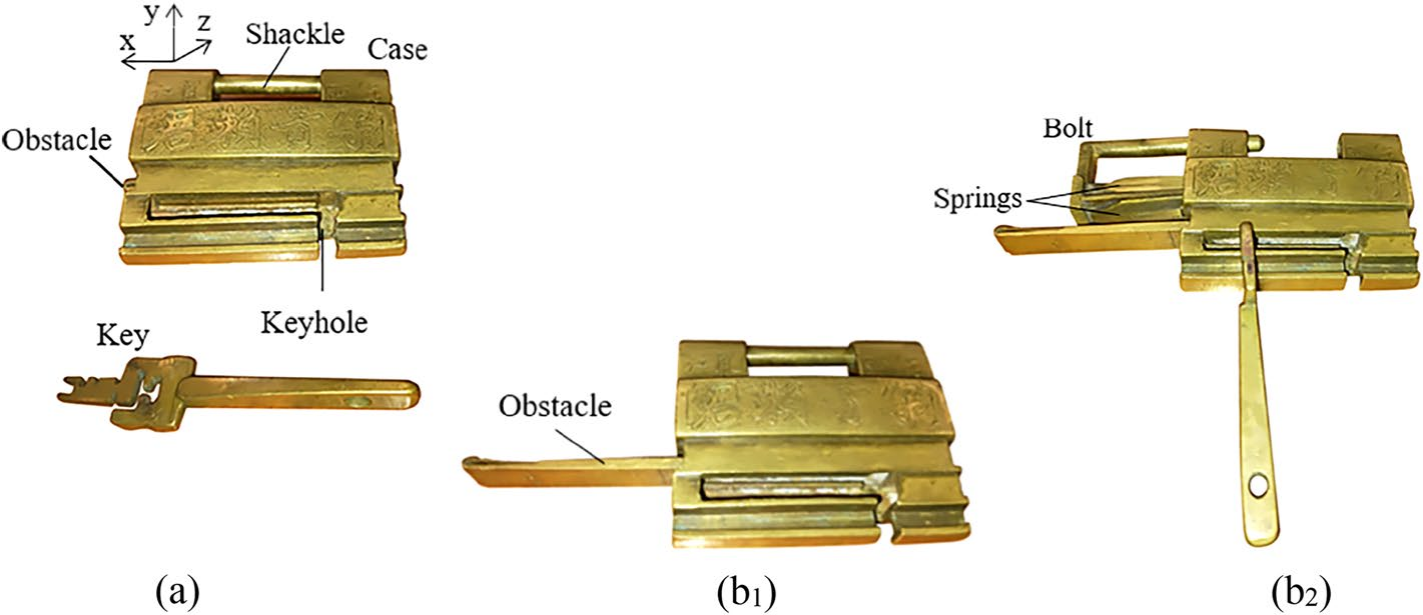
Additional obstacle lock.

Figure 2a shows an additional obstacle lock. The keyhole is located at the bottom of the case. There is an additional obstacle in the case without showing any trace to keep the key from entering the case. If the obstacle is not removed, the key cannot be inserted into the keyhole to unlock it. The members include the case, the bolt, the springs, the obstacle, and the key. When opening, the obstacle must be pulled out along the positive x-axis (Fig. 2b_1_) first. Then, the key can be inserted into the keyhole, the springs can be compressed, and the bolt can be removed to complete the unlocking (Fig. 2b_2_).

**Figure 3:**
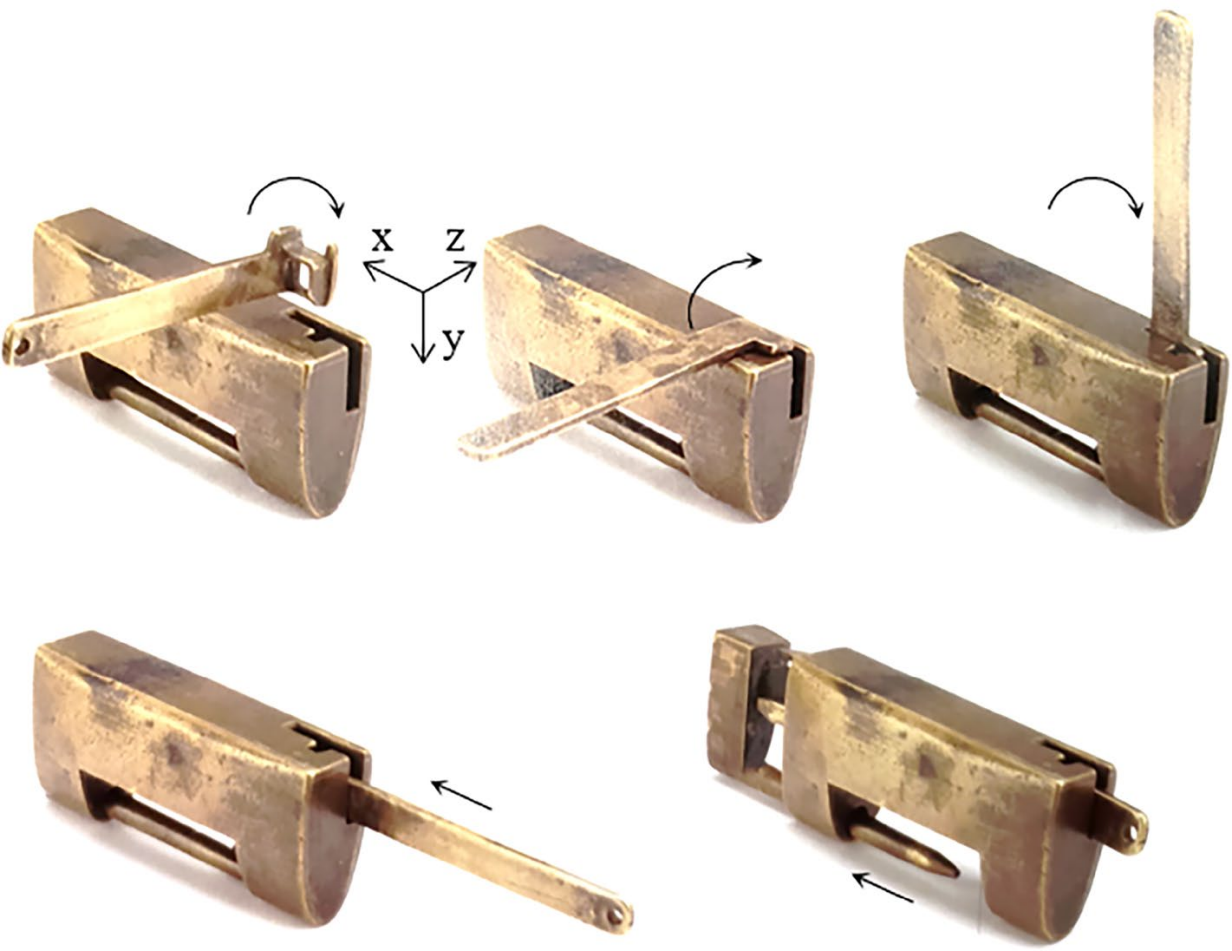
Maze lock.

Figure 3 presents a maze lock that is also called the directional lock^14^. During the unlocking process, a specific part of the key head must be contacted with a specific position of the keyhole in a specific orientation, and the key can be inserted in a specific motion. Since the process of unlocking is like walking through a maze, it is named after the maze lock. When opening, the key head is inserted into the keyhole by way of rotating it three times. The key head is rotated about the positive z-axis, the positive x-axis, and the positive z-axis with respect to the case. Then, the key is translated along the positive x-axis to compress the springs and to remove the bolt.

**Figure 4:**
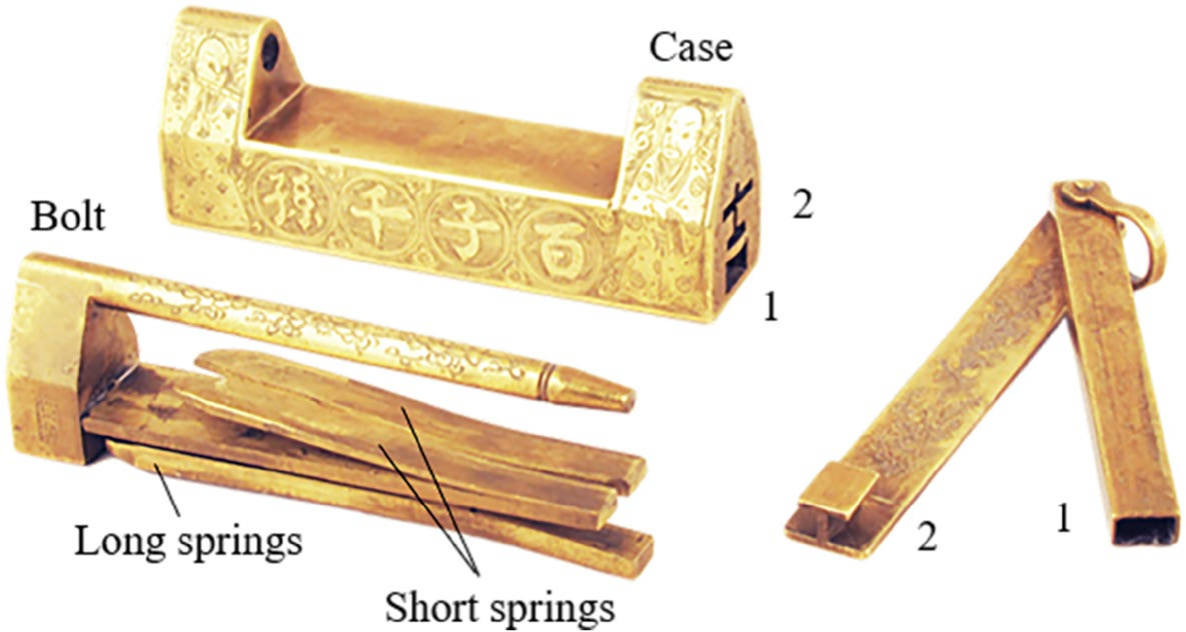
Two-section lock.

Figure 4 presents a two-section lock with two keys and two keyholes, which are divided into the upper and lower keyholes. The members include the case, the bolt, the long springs, the short springs, key 1, and key 2. When opening, key 1 is inserted into keyhole 1, the long springs are compressed, part of the bolt can be removed, and key 1 is drawn out. Next, insert the toothed pin of key 2 upward into keyhole 2, press the short springs, and remove the whole bolt to complete the unlocking.

**Figure 5:**
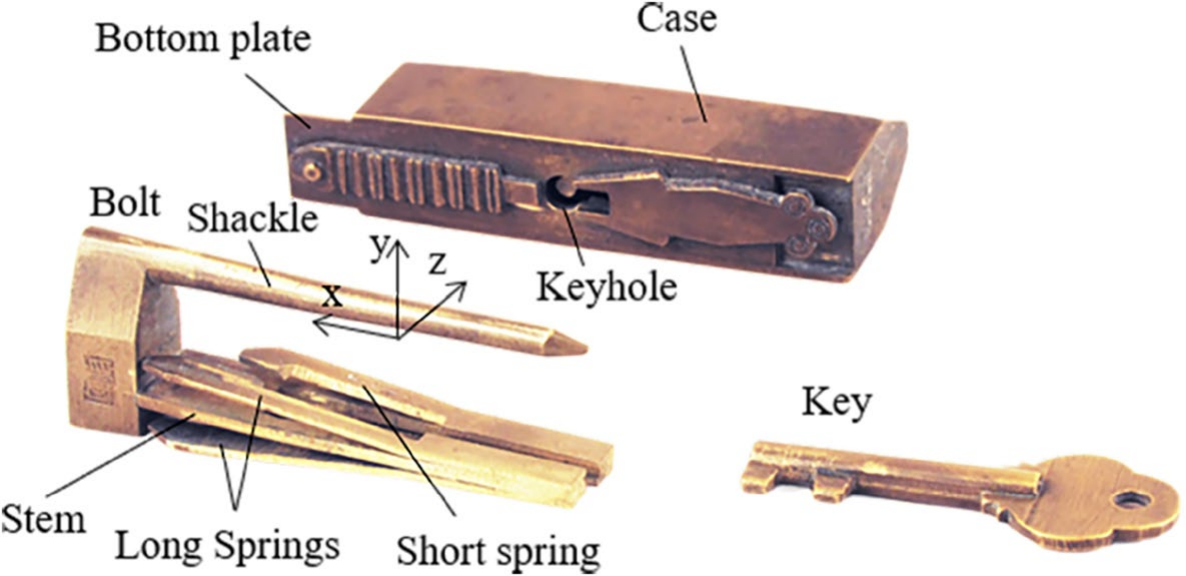
Hidden-keyhole lock.

Figure 5 presents a hidden-keyhole lock with a bottom plate for hiding the keyhole. The members have the case, the bolt, the long springs, the short springs, the bottom plate, and the key. When unlocking, the bottom plate must first move along the positive x-axis and find the keyhole. Then, the key is inserted along the positive y-axis and rotated about the negative y-axis to compress the long springs, and remove a portion of the bolt. Then, the key is rotated about the positive y-axis, it compresses the short spring, so as to remove the whole bolt and completes the unlocking.

## Representation of the topology matrix

The topological structure of a device is defined by its types and numbers of links and joints and the incidences between them^24–26^. A mechanism that has various topological structures during operation, it is called a reconfigurable mechanism^27,28^. Since the opening process of the puzzle lock has various topological structures, it belongs to a reconfigurable mechanism. The topology matrix is a valuable instrument that can be used for illustrating the topological structures of reconfigurable mechanisms, such as puzzle locks. When the number of members of a puzzle lock is N, then it is a matrix of order N^29–31^. The diagonal component n_ii_ is the type of the member i. The component n_ij_ above the diagonal is the type of the pair generated by members i and j. The component n_ji_ below the diagonal is the sequence number of the steps that occurs during operation. When the number is 1, it represents that the corresponding two members are joined first when operating the puzzle lock. When there are two or more numbers that are the same, it signifies they are moving together. If members i and j have no relative motion or are not connected, n_ij_ = n_ji_ = 0^32,33^.

Through the study of the types of pairs in puzzle locks, four types can be found during operation, including the prismatic pair (P), the revolute pair (R), the spring pair (SR), and the direct contact pair (DC). So as to illustrate the characteristics of these kinematic pairs, a symbol of such kinematic pairs that shows in the opening process can add ± β in the subscript, in which β clarifies the motion direction of the pair. Subscripts + and − illustrate the positive and negative direction of axes in the appended rectangular coordinate, respectively. It is defined that the direction of sliding out the bolt is positive x-axis, the y-axis going straight up, and the z-axis is in the light of the right-hand rule^15^. Figure 1 presents an open-keyhole lock. When the key is inserting into the keyhole, the pair incident to the key head and the case is a prismatic pair, presented as P_+x_, indicating that the key head is moved along the + x axis relative to the case (Fig. 1a). In order to put the key head in the keyhole totally, the key head must be rotated about the negative y-axis relative to the case and can be presented as R_-y_ (Fig. 1b). Then, the key can be translated in the case and the homologous pair is denoted as P_+x_ (Fig. 1c). The springs are located on the stem with a pair that can be explained as a spring pair, indicated as SR. The motion between the spring and the stem can be considered as a plane rotation. Figure 1a presents two sets of the springs that can rotate about the y and z axes and can be denoted as SR_y_ and SR_z_, respectively. Since the dimension of the springs is the same, it can be considered as one member. If the length of two sets of the springs is different, they should be considered as two members. If the motion orientation of the pair does not affect the opening process, subscripts + and − can be neglected. Direct contact pair is presented as DC that means two members are momentarily fastened by an applied force.

## Structural analysis

The complex puzzle lock skillfully hides the keyhole by using different designs, such as sliding plate, rotating disc, and pressing spring. In the process of unlocking, the position of the keyhole must be found before the key can be inserted. For this special design, how to find the position of the keyhole is a challenge. After finding the keyhole, how to insert the key head into the keyhole requires a certain technique. Furthermore, even if you can insert the key into the keyhole, you must know how to turn the key. In addition, decorative buttons or swords are added to the case. Besides adding artistic beauty, it can also be the first clue to unlocking it. The complex puzzle locks can be divided into four types, including the sliding end-plate lock, the compressing spring lock, the rotating ornament lock, and the prying bottom-plate lock. The unlocking process can be separated into two phases, the first phase of finding the keyhole, and the second phase of inserting the key, compressing the springs and removing the bolt, which is explained as follows:

### Sliding end-plate lock

**Figure 6:**
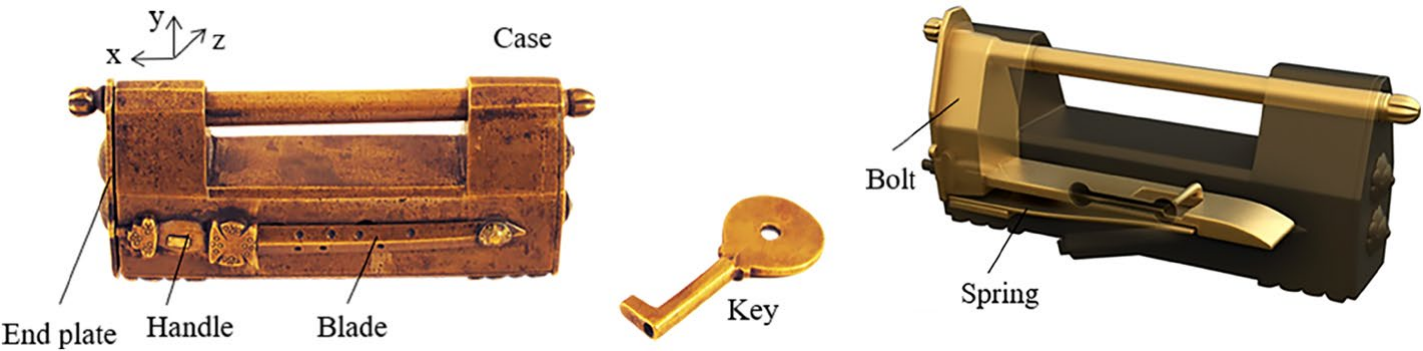
Sliding end-plate lock.

A common design of a complex puzzle lock is using a sliding plate, as the first step in unlocking, and then by adding other members, complicated unlocking modes are generated. According to the position of the plate on the case, it can be subdivided into four types: sliding bottom-plate, inner-end-plate, end-plate, and front-button. The sliding end-plate lock is an example of the unlocking process, as shown in Fig. 6.

The lock has end plates on the left and right, one end plate can be moved, the other end plate is fixed, and there is a sword on the front, which is divided into a handle and a blade. The keyhole is located behind the sword, which cannot be moved because the sword is in close contact with the movable end plate, so the keyhole is cleverly hidden. The characteristic of this lock is that the fixed end of the spring is directly connected to the bottom of the case, instead of the stem, and the opening end of the spring will jam the bolt to form a locked state. The members include the case, the bolt, the end plate, the handle, the blade, the spring, and the key.

#### Phase 1

**Figure 7:**
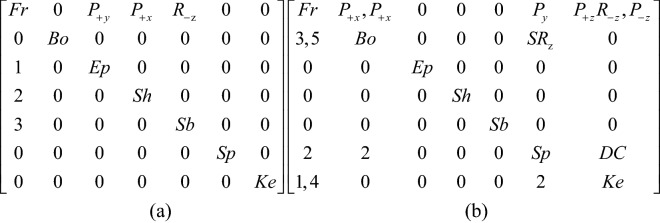
Topology matrix of sliding end-plate lock.

**Figure 8:**
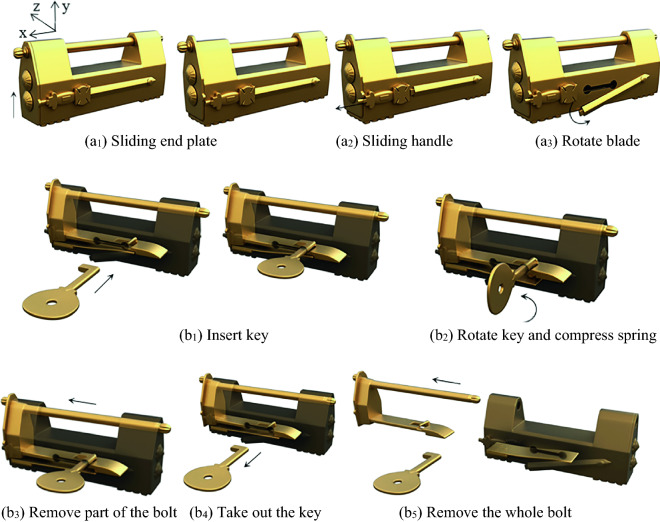
Simulation of sliding end-plate lock.

When unlocking, the first step is to slide the end plate upward, and the end plate is then connected with the case by the pair P_+y_. As the end plate that originally blocks the sliding of the handle has been removed, the handle can be moved to the outside along the positive x axis, and the handle is connected with the case by the pair P_+x_. In the third step, the keyhole can only be found by rotating the blade about the negative z axis. The corresponding topology matrix and 3D simulation are shown in Figs. 7a and 8a, respectively.

#### Phase 2

The key is inserted into the keyhole along the positive z axis, and then rotates about the negative z axis. The key in this state is connected with the case by the pair P_+z_R_-z_. At this time, the pin of the key just compresses the spring at the bottom of the case, so that the spring is separated from the bolt. Both ends of the spring are connected with the bottom of the case and the bolt by the pair P_y_ and the pair SR_z_, respectively. The key and the spring are connected by a direct contact pair DC. At this time, the spring is a ternary member. The spring blocking the movement of the bolt has been untied, so that the bolt can be moved out of a small part in the x-axis, and the bolt is connected with the case by the pair P_+x_. After removing part of the bolt, because the hollow part of the key is inserted on the stem, the movement of the bolt will be stuck, so the key must be taken out first, and the key in this state is connected with the case by the pair P_-z_. Finally, the whole part of the bolt can be removed to unlock it, and the bolt is connected with the case by the pair P_+x_. The corresponding topology matrix and 3D simulation are shown in Figs. 7b and 8b, respectively.

### Compressing spring lock

**Figure 9:**
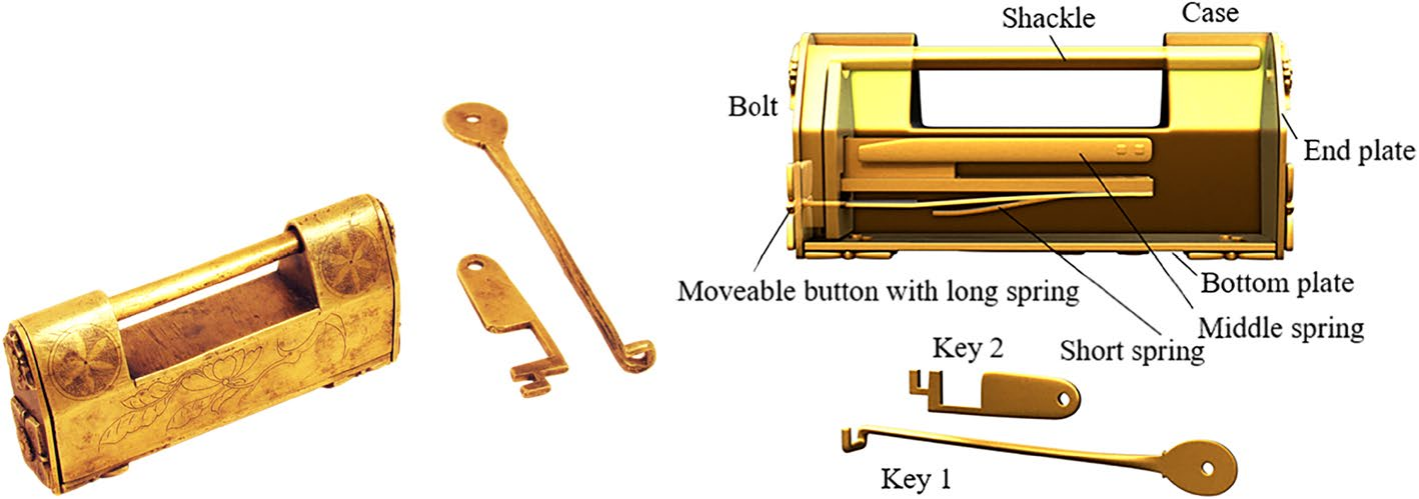
Push up end-button lock.

The barbed-spring lock is the most representative one in ancient China. Different lock forms are designed by using a change of spring inside the case. Furthermore, there are also compressing spring locks that have both an aesthetic feeling and unlocking prompt through the decorative buttons outside the case and the springs inside the case. Most of the compressing spring locks are designed with end buttons, combined with long springs, as shown in Fig. 9, which is to push up the end-button lock. The right end plate of this lock can rotate, and the left end plate has a movable button that is directly connected with the internal long spring. The bottom has a movable bottom plate, a convex point of the movable bottom plate is just inserted into the movable end plate to form a mutually fixed state, and the two keyholes are hidden. The lock stem is provided with the long, medium and short springs. The long spring contacts with the inner wall to form a locking state. The members comprise the case, the bolt, the end plate, the bottom plate, the movable button with long spring, the middle spring, the short spring, key 1, and key 2.

#### Phase 1

**Figure 10:**
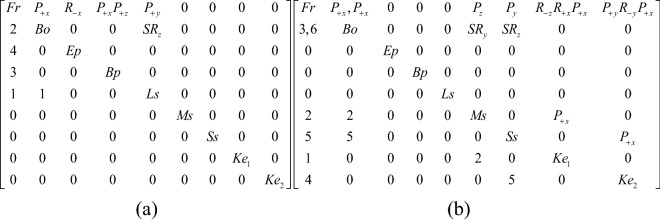
Topology matrix of push up end-button lock.

**Figure 11:**
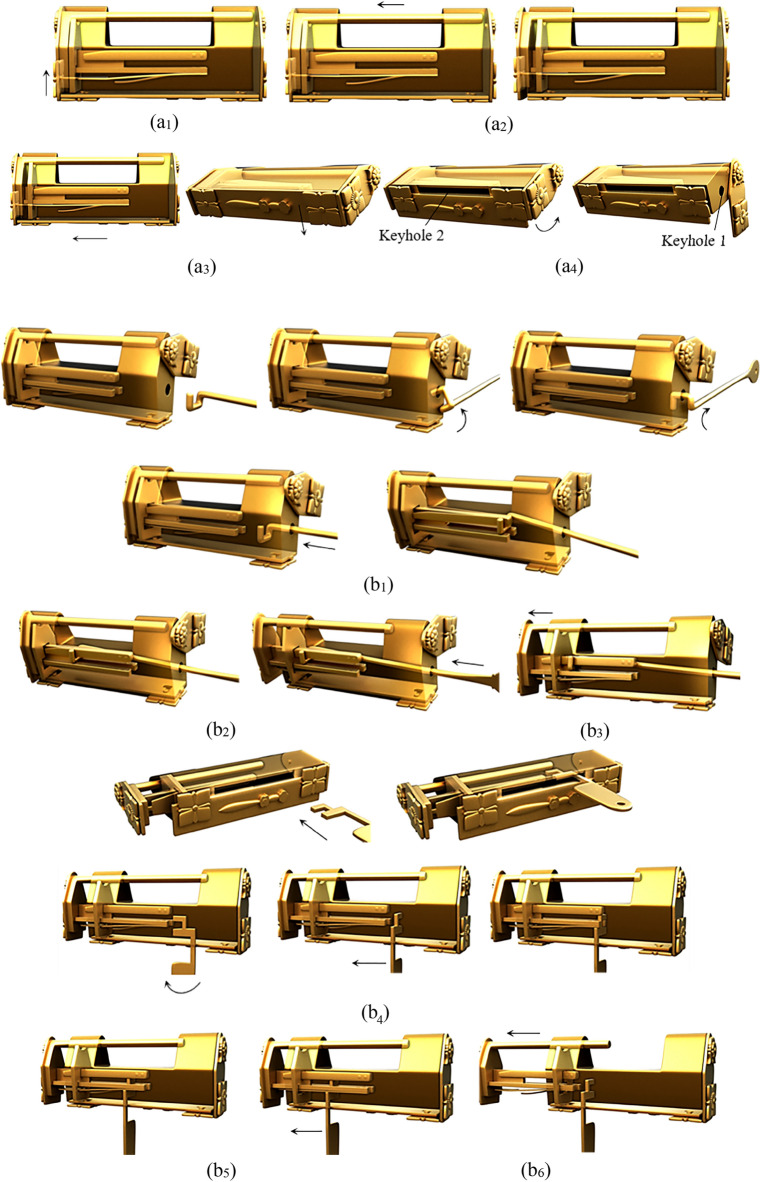
Simulation of push up end-button lock.

When unlocking, the first step is to push the movable button upward to separate the long spring from the inner wall. One end of the long spring of the movable button is connected with the case by the pair P_+y_, and the other end is connected with the bolt by the pair SR_z_. The second Step is to move the bolt to the outside along the positive x axis, and connect the bolt with the case by the pair P_+x_. As part of the bolt is removed, the bottom plate can move along the positive x axis and the positive z axis, and keyhole 2 is found. As the end plate can only rotate after the bottom plate moves, it is found that keyhole 1, the end plate and the case are connected by the pair R_-x_, and the corresponding topology matrix and 3D simulation are shown in Figs. 10a and 11a, respectively.

#### Phase 2

Firstly, key 1 is inserted into keyhole 1 by way of rotating it twice and sliding it once, and key 1 is connected with the case by the pair R_-z_R_+x_P_+x_. When key 1 compresses the middle spring, key 1 is connected with the middle spring by the pair P_+x_, and the middle spring is connected with the case and the bolt by the pair P_z_ and the pair SR_y_, respectively. After the middle spring is separated from the inner wall, a part of the bolt can be moved out and connected with the case by the pair, P_+x_. Key 2 is inserted into keyhole 2 by sliding it twice and rotating it once, and key 2 is connected with the case by the pair P_+y_R_-y_P_+x_. When key 2 presses the short spring, key 2 is connected with the short spring by the pair P_+x_, and the short spring is connected with the case and the bolt by the pair P_y_ and the pair SR_z_, respectively. When the spring is separated from the inner all, the bolt can be completely removed and connected with the case by the pair P_+x_, and the corresponding topology matrix and 3D simulation are shown in Figs. 10b and 11b, respectively.

### Rotating ornament lock

**Figure 12:**
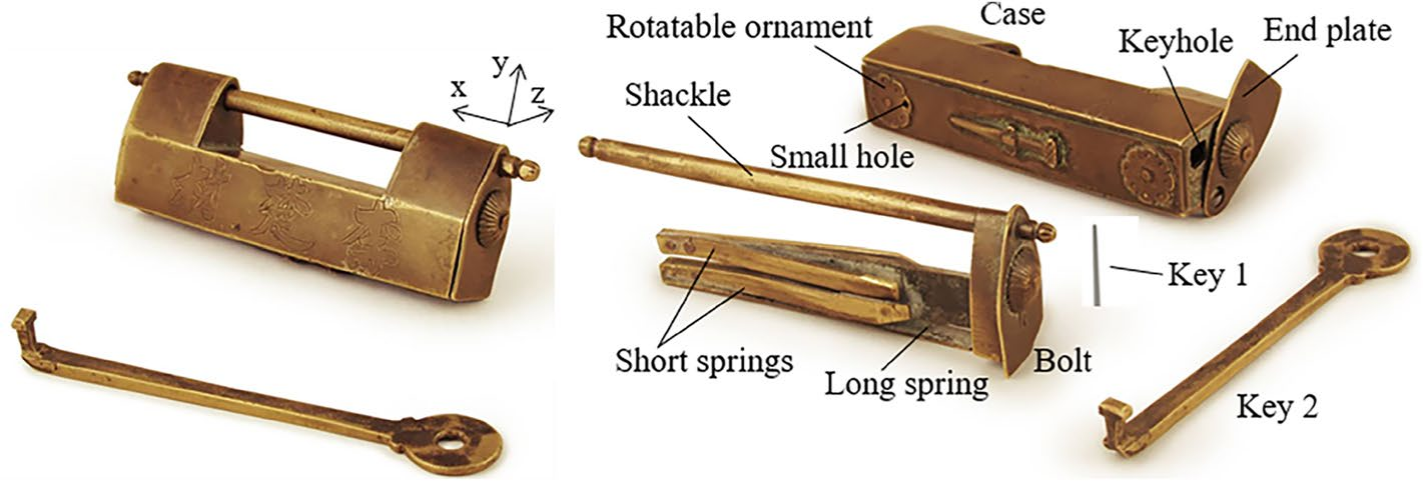
Rotating ornament lock.

Adding ornaments to the case not only increases the overall aesthetic feeling and shows the locksmith's technique, but it also serves as the first clue to unlocking it. Figure 12 shows a rotating ornament lock with a movable and fixed ornament at the bottom. The ornament consists of eight petals, each of which has a small dot. The keyhole is located on the end face and covered by an end plate, which is buckled by the end of the shackle, so that the end plate cannot rotate, thus tightly hiding the keyhole. There are five long and four short springs; the long spring is located below the stem, the four short springs are distributed on the left and right sides of the stem, the rotatable ornament at the bottom covers a small hole, and the long spring behind the small hole resists the inner wall of the lock, so that the bolt cannot be moved out and forms a locked state. The members include the case, the bolt, the long spring, the short spring, the end plate, the rotatable ornament, key 1, and key 2.

#### Phase 1

**Figure 13:**
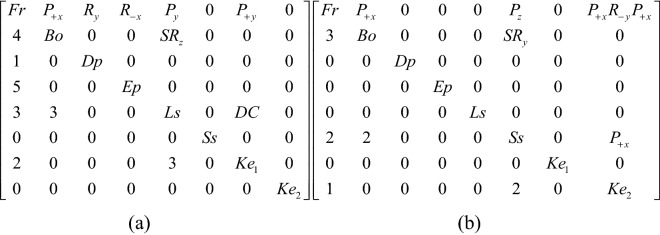
Topology matrix of rotating ornament lock.

**Figure 14:**
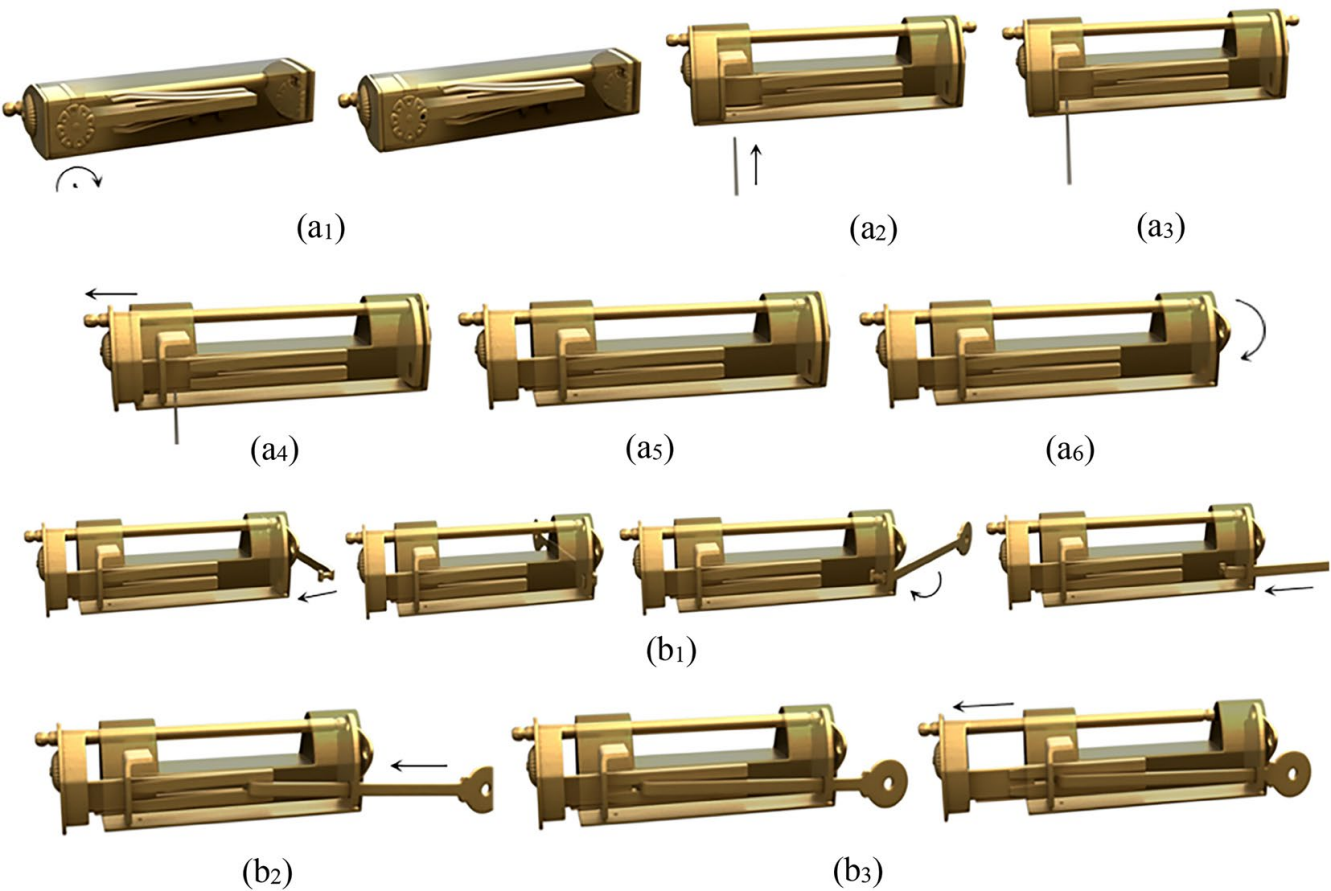
Simulation of rotating ornament lock.

When opening, the first step is to rotate the rotatable ornament until a small hole at the bottom is found, and the rotatable ornament is connected with the case by the pair R_y_. Key 1 is inserted into a small hole along the positive y axis and connected with the case by the pair P_+y_. After key 1 is inserted, the long spring is compressed, key 1 is connected with the long spring by the pair DC, and the long spring is connected with the case and the bolt by the pair P_y_ and the pair SR_z_, respectively. Due to the compression of the long spring, the bolt can move along the x-axis. Due to the removal of a portion of the bolt, the end plate can rotate about the negative x-axis to find the keyhole. The corresponding topology matrix and 3D simulation are shown in Figs. 13a and 14a, respectively.

#### Phase 2

The key 2 horizontally faces the keyhole and is inserted into the keyhole by sliding it twice and rotating once, and key 2 is connected with the case by the pair P_+x_R_-y_P_+x_. Finally, slide key 2 along the positive x-axis, press the short springs, and remove the bolt to complete the unlocking. The corresponding topology matrix and 3D simulation are shown in Figs. 13b and 14b, respectively.

### Prying bottom-plate lock

**Figure 15:**
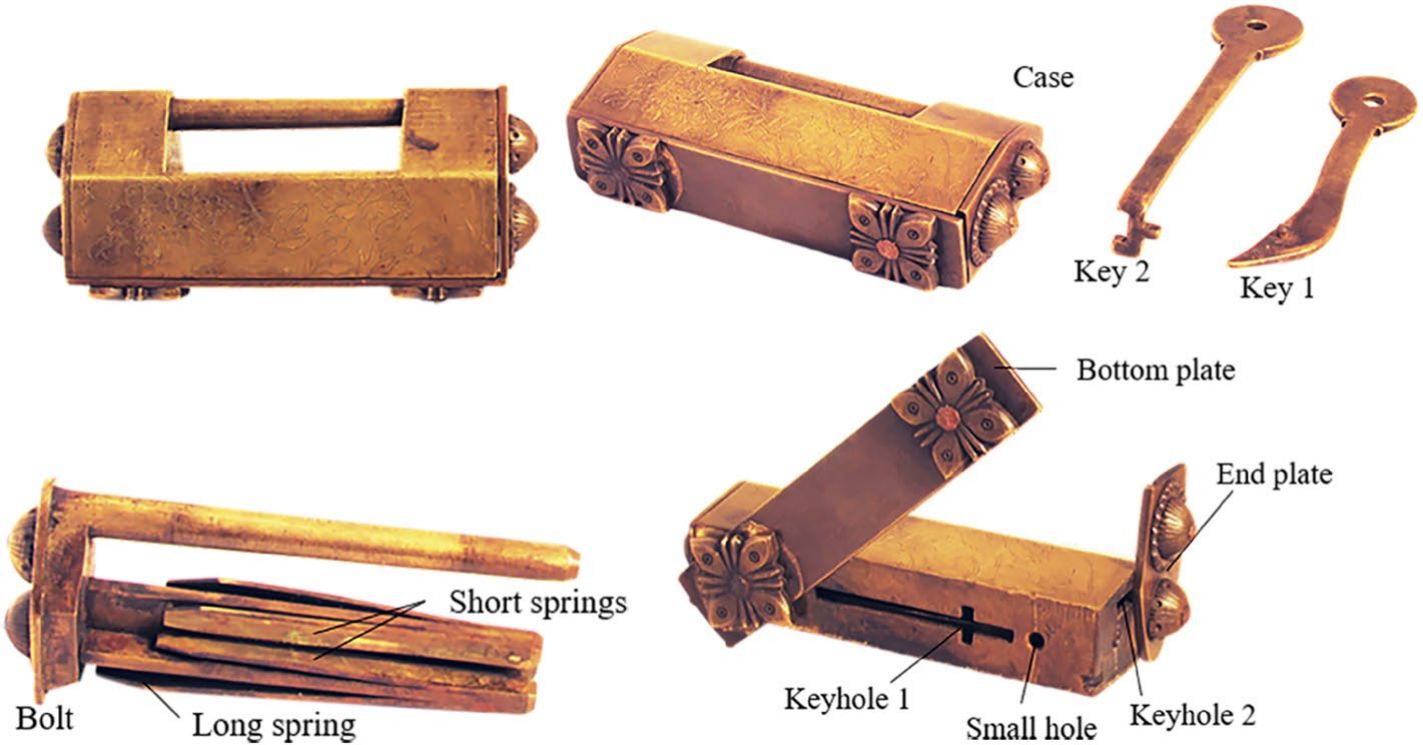
Prying bottom-plate lock.

Figure 15 shows a lock with fingers to open the bottom plate. The case is provided with exquisite and fixed decorative buttons. There are two sets of springs. The long spring supports the inner wall of the case to produce the locking effect. Two keyholes are hidden by the bottom plate and the end plate, respectively. The members include the case, the bolt, the bottom plate, the end plate, the long spring, the short springs, key 1, and key 2. There is a bump inside one side of the bottom plate, which just clamps into a small hole under the case when locking, so that the bottom plate cannot move and the keyhole is hidden.

#### Phase 1

**Figure 16:**
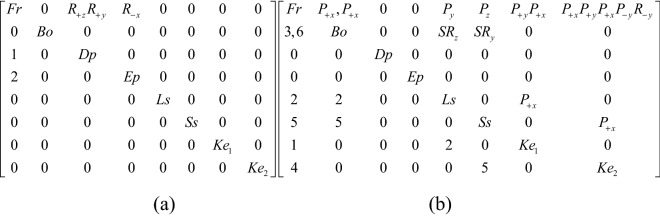
Topology matrix of prying bottom-plate lock.

**Figure 17:**
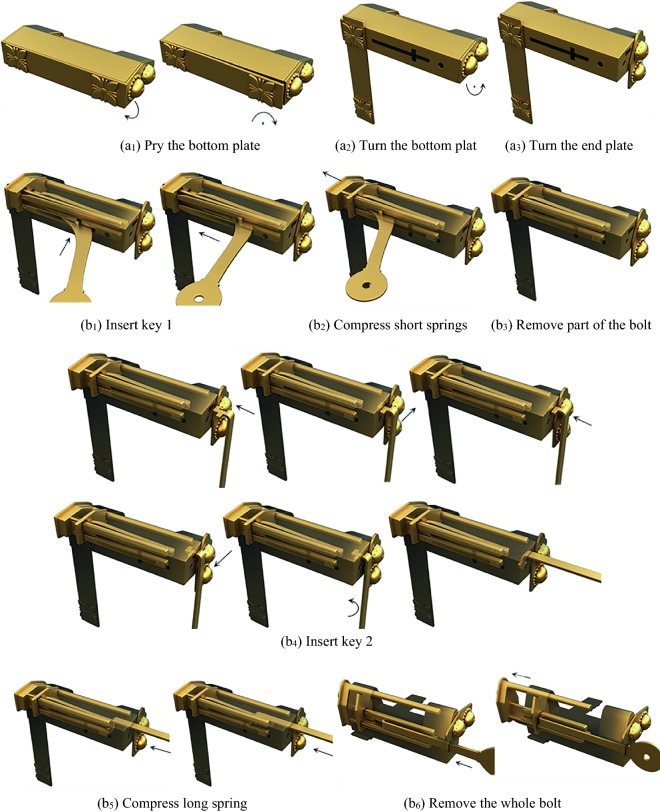
Simulation of prying bottom-plate lock.

When opening, the first step is to pry the bottom plate and then rotate it to find keyhole 1, and the bottom plate is adjacent to the case by the pair R_+z_R_+y_. Turn the end plate to find keyhole 2. The end plate and the case are connected by the pair R_+x_, and the corresponding topology matrix and 3D simulation are shown in Figs. 16a and 17a, respectively.

#### Phase 2

Key 1 is inserted into keyhole 1 by sliding it twice and is connected with the case by the pair P_+y_P_+x_. Then, slide key 1 along the positive x-axis, compress the long spring, and remove part of the bolt. Key 2 is inserted into keyhole 2 by sliding it four times and rotating once, and key 2 is connected with the case by the pair P_+x_P_+y_P_+x_P_-y_R_-y_. Then, slide key 2 along the positive x-axis, press the short springs, remove the whole bolt, and complete the unlocking. The corresponding topology matrix and 3D simulation are shown in Figs. 16b and 17b, respectively.

## Conclusions

Although a lock is a simple mechanical device, it is widely used to defend people's safety and privacy, and it is related to people's life and death. Through continuous research and exploration, ancient craftsmen have developed various types of complex puzzle locks, which belong to reconfigurable mechanisms. Their mechanical structure and design principles are very ingenious, which is the result of ancient craftsmen accumulating work experience and creative ideas in the repeated design and manufacture of locks. In this paper, the topology matrix representation method is proposed to analyze the structure of the complex puzzle lock. It is a useful tool to understand the operation of each step and the adjacency and attachment relationship between the links and the joints. According to the different opening modes, the complex puzzle locks can be divided into four types: sliding end-plate lock, compressing spring lock, rotating ornament lock, and prying bottom-plate lock. The above four locks are taken as examples to illustrate the proposed method. It provides a sketch for people to comprehend the characteristics of Chinese complex puzzle locks. From the perspective of modern mechanical science, this method of topological matrix not only clearly shows the structural changes of the puzzle lock during the unlocking process, but it is also an important segment in the restoration of other ancient machinery. Based on the result of the topology matrix, structure synthesis of the puzzle lock can be studied and more practical lock designs can be derived.

## Data Availability

No data sets and materials were used in this article.
